# Coping with Breast Cancer: A Meta-Analysis

**DOI:** 10.1371/journal.pone.0112733

**Published:** 2014-11-25

**Authors:** Pia Kvillemo, Richard Bränström

**Affiliations:** 1 Department of Clinical Neuroscience, Karolinska Institutet, Stockholm, Sweden; 2 Department of Clinical Neuroscience & Department of Public Health Sciences, Karolinska Institutet, Stockholm, Sweden; Harbin Medical University, China

## Abstract

**Objective:**

The primary aim of this study was to examine the associations between different types of coping and psychological well-being and physical health among women with breast cancer. A second aim was to explore the potential moderating influences of situational and measurement factors on the associations between coping and psychological well-being and physical health.

**Methods:**

On 14 February 2011, a literature search was made for articles published in the PubMed and PsycINFO databases before January 2010. On 5 September 2013, a repeated literature search was made for articles published before May 2013. In the final analyses, 78 studies with 11 948 participants were included.

**Results:**

Efforts to facilitate adaptation to stress, such as Acceptance and Positive Reappraisal, were related to higher well-being and health. Disengagement and avoidance types of coping were associated with lower well-being and health. The analyses indicated that, in several circumstances, coping effectiveness was dependent on cancer stage, treatment, disease duration, and type of coping measure.

**Conclusions:**

Use of coping targeting adjustment and avoiding use of disengagement forms of coping were related to better psychological well-being and physical health. Adaptive strategies and avoiding disengagement forms of coping seemed particularly beneficial for women undergoing treatment.

## Background

In 2012, almost 1.7 million women were diagnosed with breast cancer [1]. Compared with other types of cancer, breast cancer affects women at a relatively young age, but – due to better treatment and an increased rate of early detection – mortality from breast cancer has decreased during the past two decades [2]. The high number of breast cancer survivors and the fact that serious psychological distress is common among cancer survivors even many years after diagnosis and treatment [3] highlight the importance of developing interventions that target psychological distress. Knowledge of effective and maladaptive strategies to cope with stressful situations and emotions in relation to breast cancer is important in the development of such interventions. Although the research field of coping with breast cancer is extensive, findings are not uniform, and variation in measures of coping, study outcomes, and overall conclusions all require further exploration.

### Stress and coping

A majority of studies of coping are based on the Stress Appraisal and Coping Model, as presented by Lazarus and Folkman [4], in which coping is defined in terms of strategies to handle demands that go beyond perceived resources. According to the model, it is appraisals of demands and resources, rather than objective characteristics of a specific situation, that lead to and direct the coping response [4]. In addition to the excessive physiological stress caused by a breast cancer diagnosis and its treatment, many women experience psychological stress in relation to worries about diagnosis and prognosis, treatment decisions, and disruption of ordinary life functions and roles [5], [6].

### Measuring coping

There are numerous ways of responding to stress, and many attempts have been made to measure the variability [7]. The most common scales used to measure coping with breast cancer are found in the COPE, the brief-COPE, the Ways of Coping questionnaire, the Mental Adjustment of Cancer (MAC) questionnaire, and the Mini-MAC. These coping scales and other similar self-report scales differ primarily with regard to the number of subscales used and how the specific sub-scales are defined. The COPE is a 52-item self-report questionnaire measuring 14 different types of coping [8]. A short version of the COPE has been developed, using only two items to assess each subscale [9]. The subscales used were renamed, and include: self-distraction, active coping, denial, substance use, use of emotional support, use of instrumental support, behavioral disengagement, venting, positive reframing, planning, humor, acceptance, religion, and self-blame. The COPE and brief-COPE have been used to assess both habitual and dispositional coping (how people react in general), and more specific coping (how people react in relation to a specified stressful encounter). By contrast, the Ways of Coping scale was designed as a process measure of individual coping in specific stressful encounters [10]. The Ways of Coping scale is based on a 66-item questionnaire assessing frequency of use of a large number of thoughts and acts in response to a specific stressful encounter or situation. It includes the following subscales: confrontative coping, distancing, self-controlling, seeking social support, accepting responsibility, escape-avoidance, planful problem-solving, and positive reappraisal. The MAC scale is a measure of coping specifically developed for cancer patients [11]. It was originally designed to assess five styles of adjustment to cancer: fighting spirit, anxious preoccupation, cognitive avoidance, helpless-hopelessness, and fatalism. A short version of the MAC has been developed with 29 items, the so-called Mini-MAC [12]. In addition to scales for the five styles, a large number of additional scales have been used to assess coping with breast cancer. To address the difficulties associated with the large number of related but discrete subscales used, it is common to form groups of subscales or factors. Common higher-order dichotomized factors presented in the literature are: problem-focused vs. emotion-focused, avoidance vs. approach, active vs. passive, engagement vs. disengagement, and cognitive vs. behavioral [7].

### Aim of the study

The primary aim of this study was to examine coping among women with breast cancer, and analyze which types of coping are related to three broad categories of outcomes: psychological well-being with both positive and negative affect, and also aspects of physical health. A second aim was to explore the potential moderating influences of situational factors (cancer stage, current treatment, time since diagnosis) and measurement factors (cancer-specific vs. dispositional coping) on the associations between coping and psychological and physical states. The moderators were selected on the basis that they reflect situations were different stressors and coping responses might be salient, and because it was possible to obtain information about them that could be compared between studies.

### Disposition

In the method section we describe the selection of articles, data extraction and the principles for classification of coping strategies and variables related to psychological or physical states. We also describe the operationalization of moderators and the statistical methods used. In the result section we present correlations between lower and higher order coping strategies and psychological or physical states and also results of the testing of different moderators. The results are discussed and conclusions are presented at the end of the article.

## Methods

**Figure 1:**
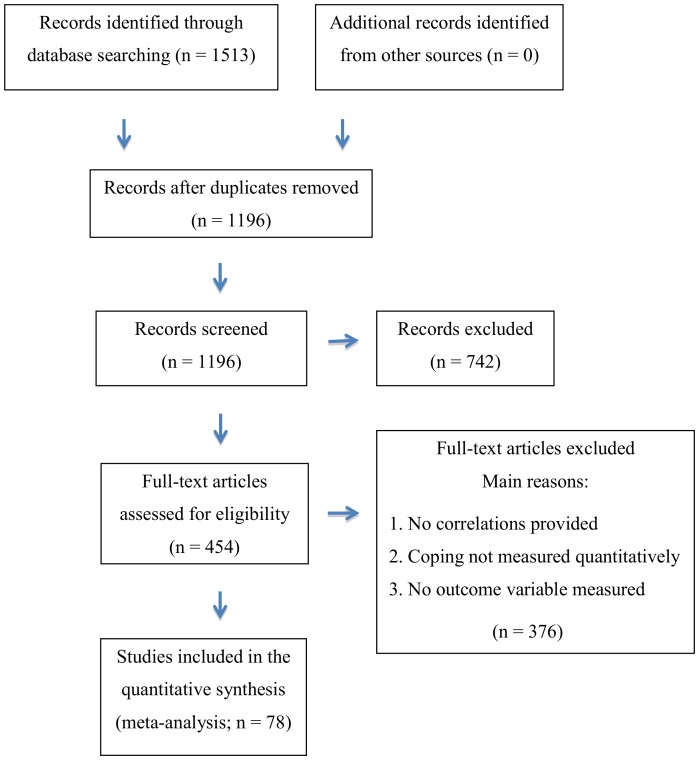
Flow diagram of the article-selection process.

On the 14^th^ of February 2011, a literature search was conducted in the PubMed and PsycINFO databases for articles published before January 2010. Keywords used were: “Breast Cancer” or “Breast Neoplasm” and “Coping”. Limits used in PubMed were “English language”, “Human subjects”, “Published 1860–2010”, “Peer-reviewed journal” and “Above 18 years”. The search resulted in 437 articles from PubMed, and 397 articles from PsycINFO. After removing duplicates, 627 articles remained. On the 5^th^ of September 2013, the same literature search was repeated with the same keywords in the same databases for articles published between January 1, 2010 and April 30, 2013. The search resulted in 476 articles from PubMed, and 203 articles from PsycINFO. After removing duplicates, 569 articles remained. The selection process and number of articles are summarized and illustrated in Figure 1.

### Ethics statement

The study concerns analyses of already collected and published data, and no ethical dilemmas arise.

### Selection of articles

The authors read all abstracts to the articles to determine possible inclusion. Inclusion criteria were: (a) published in peer-reviewed scientific journals; (b) written in English; c) women diagnosed with breast cancer; (d) participants 18 years or older; (e) from the United States, Canada, Europe, or Australia/New Zealand; (f) coping measured quantitatively; (g) sample size larger than n = 30; and (h) at least one quantitatively measured psychological state or aspect of physical health. Publication in peer-reviewed scientific journals means that the articles have been examined by researchers in the field and found to meet certain scientific requirements. Exclusion of studies in other languages than English was due to practical reasons as we regarded translations of articles too time consuming. The reason for exclusion of studies from non-Western countries was that the current coping-scales have mostly been developed and used in the West, and we do not know how the scales are perceived in different cultures and how different perceptions might affect the result [13]. During the screening 742 articles were excluded. The remaining articles were read in full by the current authors. In order to be included, the studies had to present zero-order (bivariate) correlations between coping style and one or more psychological or physical states. If the same sample was used in more than one article, the article that provided the greatest number of correlations was included. When correlations were not included in the article, the primary author was contacted to ask whether the relevant data could be obtained. 104 authors were contacted, and 15 of them provided useful data (Table 1, nos: 1, 9, 13, 15, 18, 28, 29, 42, 51, 52, 54, 56, 63, 69, 74). The most common reasons for excluding an article, whether excluded at the stage of abstract assessment or in connection with the full text assessment, were that no correlations were provided, that coping was not measured quantitatively, and that no psychological or physical variable was measured. The articles were often put aside as soon as the authors discovered that any of the inclusion criteria was missing, but sometimes it was immediately obvious that there were many criteria not met in the current article.

**Table 1:** Description of studies included in the meta-analysis.

Art.no	Author	Year	N	Disease status	Stage	Mean Age	Mean years since diag.	Coping measure	Cancer-specific.	Types of coping^a^	Health rel. var.
1	Alder	2003	126	First diag.	Not stat.	61	2.7	FQC	Yes	DA; Ru; Sp; Ho; EA/Di; SC; FS	2 NA
2	Anagnostopoulos	2010	153	First diag.	0–III	58	5.2	Mini-MAC	Yes	FS; Ho; Ru; BD; EA/Di	PA; NA
3	Andreau	2012	102	First diag.	l–lll	51	<0.2	Mini-MAC	Yes	Ho, Ru, FS, EA/Di, BD	NA
4	Arathuzik	1991	80	Not stat.	III–IV	Not stat.	Not stat.	The Pain Coping Tool	No	SSS; DA; EA/Di; ADD; Acc; PR	3 NA; PH
5	Astin	1999	58	First diag.	0–III	54	0.1	Shapiro Control Inventory	No	DA; Acc; Ho	2 NA; PH
6	Aukst-Margetic	2009	115	Not stat.	I–IV	62	Not stat.	Three separate items	Yes	Sp	PA; PH
7	Bellizzi	2006	224	First diag.	I–IV	60	Not stat.	Brief COPE	Yes	DC	PA
8	Bigatti	2012	65	Mixed	0–lV	52	3.1	WOC	Not stat.	SC, Pl, SSS, SB, CC, PR, EA/Di	NA
9	Boehmer	2011	112	First diag.	l–lll	55	6.4	Mini-MAC	Yes	FS, Ru, BD, EA/Di, Ho	PA; 2 NA; PH
10	Broeckel	1998	61	First diag.	I–III	52	1.8	Fatigue Catastrophizing Scale	No	Ho	PH
11	Buddeberg	1991	107	First diag.	I–IV	53	0.5	FQC; Zurich Coping Questionnaire	Not stat.	Ru; FS; EA/Di	2 PH
12	Bussell	2010	59	Mixed	DCIS-VI	50	Not stat.	Brief COPE	Yes	EA/Di; DA; ADD; SSS; BD; V; PR; Pl; Acc; Sp; SB	4 NA; 2 PH
13	Carlsson	2001	120	Mixed	Not stat.	49	Not stat.	MAC	Yes	FS; Ho; Ru; BD; EA/Di	PA
14	Carver	1993	52	First diag.	I or II	58	<0.1	COPE	Yes	DA; EA/Di; SC; Acc; BD; Pl; SSS; PR; Sp	NA
15	Charlier	2012	464	Mixed	Not stat.	53	Not stat.	CISS-NL	No	DA; SSS, EA/Di	3 NA; 4 PH
16	Compas	1999	80	Not stat.	I–IV	55	<0.1	CSI	Yes	DA; PR; SSS; V; Ho; EA/Di; SB; 17SI	2 NA
17	Compas	2006	232	First diag.	0–III	52	0.3	RSQ-CV	Yes	P18CC, SCC, DC	NA
18	Danhauer	2009	246	First diag.	l–III	43	0.6	WOC	Yes	SSS; EA/Di; PR; Ho; DA; Sp; SC	PA; PH
19	Dasch	2010	53	Not stat.	0–IV	53	0.1	Brief COPE	Yes	EA/Di; DA; ADD; SSS; BD; V; PR; Pl; Acc; Sp; SB	NA
20	David	2006	60	First diag.	Not stat.	52	Not stat.	Brief COPE	Yes	EA/Di; DA; ADD; SSS; BD; V; PR; Pl; Acc; Sp; SB	NA
21	Dedert	2012	75	Mixed	l–lV	52	<0.1	Brief COPE	Yes	EA/Di	PH
22	Epping-Jordan	1999	80	Not stat.	I–IV	55	<0.1	CSI	Yes	DA; DC	NA
23	Filazoglu	2008	188	First diag.	I–IV	45	0.6	WOC	Not stat.	Ho; DA; Sp; PR	PA; PH
24	Fillion	2002	132	First diag.	Not stat.	54	<0.1	Brief COPE	No	DA; EA/Di; SSS; ADD; Sp	PA; 2 NA; PH
25	Fillion	2003	277	First diag.	I–III	54	1.1	CHIP	Yes	DA; DC	PH
26	Fischer	2013	57	First diag.	Not stat.	51	Not stat.	COPE	Yes	Acc, DA, EA/Di, SSS	2 NA
27	Gall	2000	32	Mixed	Not stat.	50	2.5	RCOPE	Yes	Sp	PA; NA
28	Gall	2009	91	Not stat.	Not stat.	61	<0.1	RCOPE	Yes	Acc; ADD; BD; DA; EA/Di; Pl; PR; SB; Sp; SSS; V	PA; NA; PH
29	Gaston	2013	73	Not stat.	ll–lV	Not stat.	Not stat.	CSQ (Coping strategy questionnaire)	Not stat.	EA/Di, PR, Sp, DA, V	PA
30	Gelinas	2004	103	First diag.	I–III	54	Not stat.	CHIP	No	DC, DA	NA; 2PH
31	Glinder	2007	135	First diag.	0–III	52	<0.3	RSQ-CV	Yes	PCC; SCC; DC	PA; 4 NA
32	Groarke	2011	241	First diag.	0–lV	53	Not stat.	CISS, MAC	Both	DA, BD; SSS; EA/Di, FS, Ru, Ho	PA 3 NA
33	Hebert	2009	284	First diag.	I–IV	51	Not stat.	RCOPE	Not stat.	Sp	2 PA; NA; PH
34	Holland	2003	56	First diag.	I or II	48	0.9	WOC	Yes	DC	PA
35	Holly	2003	64	Not stat.	Not stat.	52	Not stat.	MAC	Yes	FS; Ho; Ru; BD; EA/Di	2 NA
36	Jadoulle	2006	151	First diag.	Not stat.	57	0	CHIP	No	EA/Di; DA; Ru	2 NA
37	Jim	2006	167	First diag.	II–III	51	<0.1	COPE	No	PR; DA; Sp; EA/Di	PA
38	Karademas	2007	103	First diag.	I–III	55	9	WOC	Yes	PR; SSS; Sp; EA/Di	3 NA; PH
39	Kershaw	2004	189	Recurrent	llI–IV	54	Not stat.	Brief COPE	No	DC	PA; 2 PH
40	Kim	2010	231	Mixed	I–IV	51	Not stat.	Brief COPE	Yes	PR, SB	PA
41	Komproe	1997	109	Not stat.	I–IV	61	<0.2	UCL	Not stat.	DA; SSS	NA
42	Lauver	2007	46	Not stat.	I–III	51	Not stat.	Brief COPE	Yes	BD; EA/Di; DA; ADD; SSS; SB; PR; Pl; Acc; Sp; V	PA; NA; PH
43	Lebel	2008	86	First diag.	I–III	62	0.2	WOC	Yes	DA; SSS; EA/Di	NA
44	Levy	1990	120	First diag.	I or II	50	<0.1	WOC	No	SSS	PH
45	Low	2006	417	First diag.	I or II	58	<0.2	COPE	Yes	FS; EA/Di, DA; PR; Sp; Acc	PA; 2 NA
46	Manne	2007	238	First diag.	0–III	49	Not stat.	Emotional Processing Scale; COPE	Yes	Acc; Ru; V	PA; 4 NA
47	Manne	1994	43	First diag.	Not stat.	48	<0.1	WOC	Yes	CC; SC; SSS; SB; EA/Di; DA; PR	PA; NA; PH
48	Manning-Walsh	2005	100	Not stat.	I–IV	46	0.8	Negative Coping subscale of RCOPE	Yes	Sp	2 PA
49	Matthews	2009	93	First diag.	I–IV	60	Not stat.	Jalowiec Coping Scale	Yes	DA	PA
50	McCaul	1999	61	First diag.	I or II	51	<0.1	Coping Response Indices	Yes	DC	PA; NA; 2PH
51	Mcorry	2012	72	Not stat.	0–lll	57	<0.5	Cancer coping questionnaire	Yes	PR, EA/Di, Pl	2 NA
52	Mehnert	2009	1083	Mixed	I–IV	62	3.9	Dealing with Illness Inventory	Yes	PR; EA/Di; DA; Sp	PA; NA; PH
53	Nelson	1989	128	First diag.	Not stat.	54	Not stat.	Form A of Health and Daily Living Form	Yes	FS; DA; EA/Di	NA
54	Northouse	2013	157	Not stat.	lll–lV	Not stat.	Not stat.	Brief COPE	Not stat.	DA; EA/Di	PA; PH
55	Osborne	1999	632	First diag.	I–II	Not stat.	<1	Mini-MAC	Yes	BD; FS; PR; Ho; Ru	2 NA
56	Puig	2006	41	Not stat.	I–II	51	<1	Emotional Approach Coping Scale	Not stat.	V	PA; 2 NA; PH
57	Ransom	2005	146	First diag.	0–II	56	Not stat.	Illness Management Questionnaire	Yes	EA/Di; DA; Ru	PH
58	Reddick	2005	138	First diag.	II–IV	45	Not stat.	Coping strategy questionnaire (CSQ)	No	EA/Di; PR; DA; Sp; Ho	2 NA; PH
59	Reid-Arndt	2012	36	Not stat.	0–lV	56	<0.1	MAC, Brief COPE	Yes	Ho, Ru, FS, DC, EA/Di, ADD	NA
60	Reuter	2006	353	First diag.	I–III	50	<1	Mini-MAC	Yes	FS; BD; Ru; Ho; EA/Di	NA; PH
61	Romero	2008	45	First diag.	I or II	51	<0.1	32-item vers.of the Coping Responses Inv.	Yes	DA; DC	NA
62	Rottman	2010	684	Not stat.	l–lll	54	1.4	Mini-MAC	Yes	BD; EA/Di, FS, Ho, Ru	PA; PH
63	Roussi	2007	72	Not stat.	Not stat.	54	<0.1	Brief-COPE	Yes	EA/Di; DA; ADD; SSS; BD; Pl; Acc; Sp; V; SC; PR	PA; NA; PH
64	Rozema	2009	119	First diag.	Not stat.	47	1.2	UCL	Not stat.	DA; BD; SSS; V; EA/Di	PA; NA; PH
65	Schlegel	2009	223	Not stat.	I–IV	59	Not stat.	COPE	No	DA; Pl; SSS; PR; Acc; Sp; Ru; BD; ADD; SC; EA/Di	NA
66	Schoen	2004	248	Not stat.	0–III	61	3.2	WOC - Cancer inventory	Yes	SSS; PR; EA/Di; BD	PA; PH
67	Sears	2003	92	First diag.	I or II	52	0.6	Positive Reappraisal subscale (COPE)	Yes	PR	3 PA; 2 NA; 2 PH
68	Silva	2012	50	First diag.	l–ll	52	1.4	Brief COPE	Not stat.	SSS	PA 2 NA
69	Smith	2011	44	Not stat.	“Advanced”	52	Not stat.	Brief COPE	Not stat.	Acc; ADD; BD; DA; EA/Di; Pl; PR; SB; Sp; SSS; V	2 PA 2 NA 2 PH
70	Stanton	2000	92	First diag.	I–II	52	0.6	COPE	Yes	Ru; V	3 PA; NA; 2 PH
71	Stanton	2012	103	First diag.	Not stat.	57	8	COPE Inventory	Yes	Ru; V	PA 2 NA
72	Taha	2012	42	Not stat.	Not stat.	44	Not stat.	SCOPE	Yes	DA, DC	NA
73	Thune-Boyle	2013	155	First diag.	l–ll	56	<1	Brief COPE	Not stat.	Acc; SSS; EA/Di; Pl; V; SB	2 NA
74	Urcuyo	2005	230	First diag.	0–II	53	<1	Brief COPE	Yes	DA; Pl; SSS; PR; Acc; Sp; V; BD; ADD; EA/Di	PA; 2 NA
75	Wade	2005	44	Not stat.	I or II	60	<0.2	WOC	Yes	EA/Di	NA
76	Vos	2004	87	First diag.	0–II	50	<1	UCL; Health and Diseases Inventories	Not stat.	SSS; PR; Ru; EA/Di	PA; NA
77	Yang	2008	65	Recurrent	Metastatic	54	<0.1	Brief COPE	No	DC	PA; 2NA
78	Zwingmann	2006	156	First diag.	0–IV	56	0.9	FQC; Adjusted Brief RCOPE	Yes	Sp; Ru; FS	2 NA

^a^The way they were classified in the coding process

*Note.* **Coping measures**: MAC  =  Mental Adjustment to Cancer (Greer & Watson, 1987); Mini-MAC  =  Mini-mental Adjustment to cancer (Watson, Law, dos Santos, Greer, Baruch, & Bliss, 1994); CISS =  Coping Inventory for Stressful Situations (Endler & Parker, 1990); CSI  =  Coping Strategies Inventory (Tobin, Holroyd, Reynolds, & Wigal, 1989); CHIP  =  Coping with Health Injuries and Problems scale (Endler, Parker, & Summerfeldt, 1998); WOC  =  Ways of Coping Questionnaire (Folkman & Lazarus, 1980); FQC  =  Freiburg Questionnaire of Coping with Illness (Muthny, 1988); RSQ-CV  =  Responses to Stress questionnaire - cancer version (Compas, et al., 2006); RCOPE  =  Religious coping scale (Pargament, Koenig, & Perez, 2000); UCL  =  Utrecht Coping List (Schreurs & van de Willige, 1988). **Types of coping**: Acc  =  Acceptance; ADD  =  Alcohol/Drug Disengagement; App =  Approach; BD  =  Behavioral Disengagement; DA  =  Direct Action; DC  =  Disengagement Coping; EA/Di  =  Escape/Avoidance/Distancing; EF  =  Emotion Focused; FS  =  Fighting Spirit; Ho  =  Hopelessness; PCC  =  Primary Control Coping; PF  =  Problem Focused; Pl  =  Planning; PR  =  Positive Reappraisal; Ru  =  Rumination; SB  =  Self-Blame; SC  =  Self-Controlling; SCC  =  Secondary Control Coping; SI  =  Social Isolation; Sp  =  Spirituality; SSS  =  Seeking Social Support; V =  Venting. **Outcomes**: NA  =  negative affect; PA  =  positive affect; PH  =  physical health.

### Data extraction

The data extracted from each article were: (a) author; (b) year of publication; (c) sample size; (d) cross-sectional study or prospective study; (e) disease status (newly diagnosed vs. recurrent disease); (f) cancer/disease stage; (g) current treatment; (h) study location; (i) mean age of participants; (j) mean years since diagnosis; (k) educational level; (l) income; (m) type of stressor addressed; (n) timeframe of stressor; (o) cancer-specific (yes/no); (p) coping scale used; (q) type of coping categories; and (r) psychological or physical states (measured at or around diagnosis; shortly after diagnosis [1–6 months]; a longer time after diagnosis [6–18 months]; at long-term follow-up [>18 months]). A correlation coefficient for each type of coping and psychological or physical states was gathered, or calculated, from each study and served as effect size. If there were cross-sectional data measured at several time-points, we chose the time-point furthest from study entry in order to get a sufficient number of studies were the correlation coefficient represented another time point than the one around diagnosis. For longitudinal studies, reporting on correlations between baseline coping and follow-up coping, prospective correlations were also gathered. If there were several prospective time-points, we chose the one furthest from the time of measuring coping. If the studies included both cross-sectional data at several time-points and prospective data, we chose to include the cross-sectional data that were obtained at the time closest to study entry, and also the prospective data. Both authors coded and verified all the variables. Disagreements regarding coping classification and which psychological and physical variables to include, were resolved by discussion.

### Coding of coping strategies

**Figure 2:**
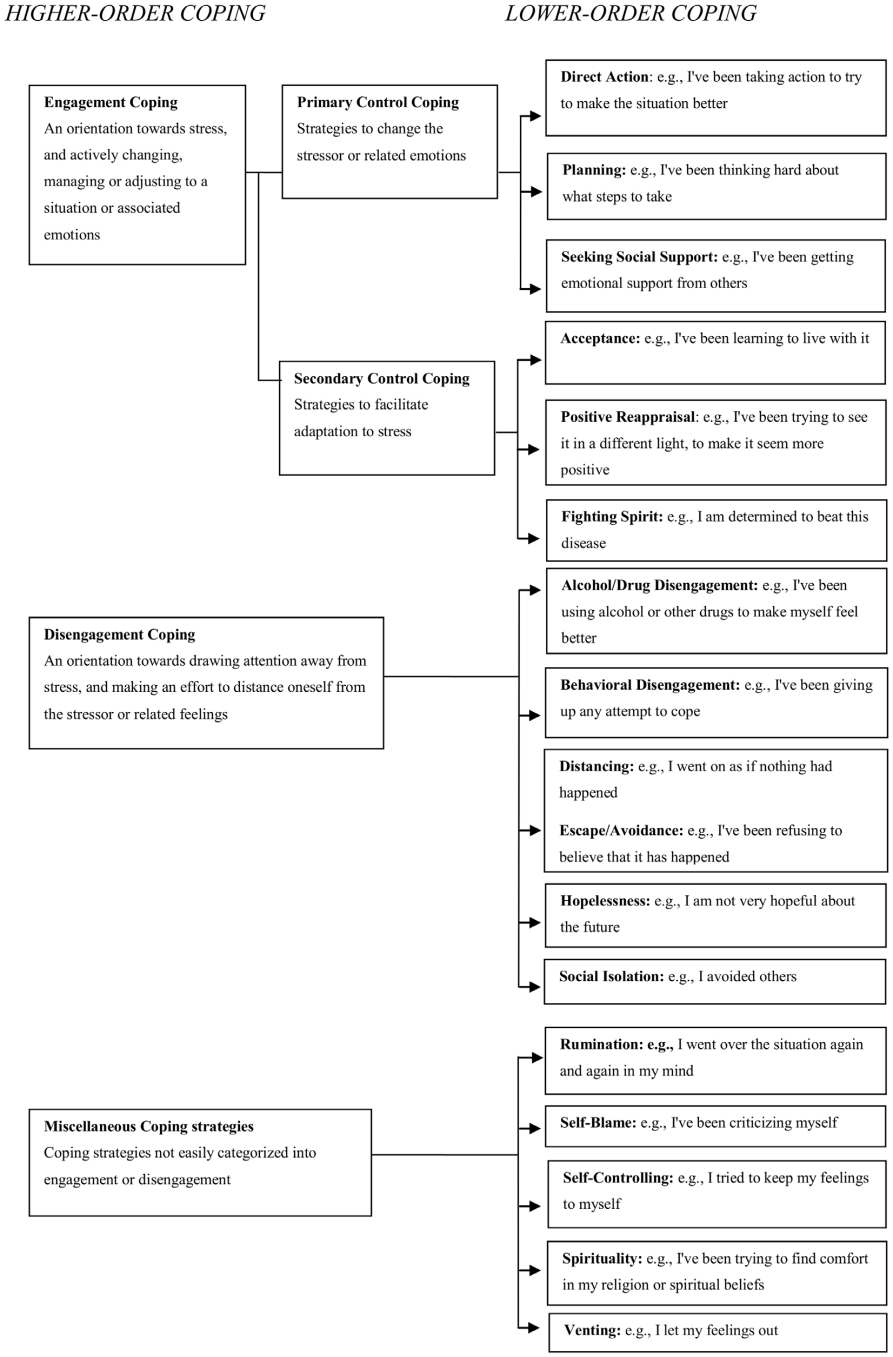
Proposed coping hierarchy and coping scales included in the meta-analysis.

Two approaches were adopted to resolve the difficulties involved in the classification of coping. The first was to use a lower-order classification based on specific subscales from the Ways of Coping scales and COPE, a procedure that was adopted in a previous meta-analysis of coping effectiveness by Moskowitz et al. [14]. In cases where measures other than Ways of Coping and COPE were used, the coping measures were placed on the scales they most closely resembled. The lower-order subcategories, Escape/Avoidance and Distancing, were put together into a common category because they had many similarities. The second approach was to use a higher-order classification based on factors previously described by Connor-Smith et al. [15] and Compas et al. [16]. In their research, they distinguish between voluntary coping responses (primary control engagement coping, secondary control engagement coping, disengagement coping) and involuntary coping responses (involuntary engagement, involuntary disengagement). Since the majority of current studies on coping do not include measures of involuntary coping [16], our higher-order categorization of coping strategies included: primary control engagement coping (PCC), secondary control engagement coping (SCC), and disengagement coping (DC). Connor-Smith et al. [15] presents a table were different higher level categories are defined. PCC is defined as “Active attempts to control or change a bad situation or one's emotional reaction to the situation”. SCC is defined as “Attempts to adapt to a stressor to create a better fit between the self and the environment”. DC, finally, represents responses that are oriented away from the stressor or one's reactions to the stressor. The different coping strategies were primarily put into the lower-order categories, but if that was not possible, they were put into a higher-order category. If a coping strategy could not be put into any of the categories, or if there were no correlations presented for that specific strategy, it was left out of the analysis. In total, 14 coping strategies from 13 articles were left out, amounting to less than 4 percent of all the coping strategies presented in the articles included. The lower-order subcategories of coping included in each higher-order category are presented in Figure 2.

### Classification of psychological and physical states

Psychological and physical states were categorized into three groups: positive affect, negative affect, and aspects of physical health. The three groups are also referred to as health-related variables [17]. The guiding principle for the selection of health related variables was to include all psychological and physical states reported, but to exclude social aspects. If correlations between coping and more than one health-related variable of each kind were reported, and no summary measure for them was presented, an average for the correlations for that category was calculated and used. Data were entered such that high scores indicated higher positive affect, higher negative affect, or higher degree of physical health. Positive affective states included: emotional/psychological well-being (Table 1, nos: 6, 18, 28, 34, 40, 54), vitality/vigor (Table 1, nos: 24, 45, 63, 67, 70, 76), quality of life (Table 1, nos: 13, 48, 50, 66, 69,74,77), mental health (Table 1, nos: 2, 9, 23, 33, 39, 46, 52, 64), life satisfaction (Table 1, nos: 7, 27,33,71), hope (Table 1, nos: 67, 70), appreciation for life (Table 1, no. 7), positive mood/affect life (Table 1, nos: 31, 32, 42, 47), emotional functioning (Table 1, no. 62) and meaning in life (Table 1, no. 37). Negative affective states included: depression (Table 1, nos: 1, 4, 5, 8, 9, 15, 16, 24, 32, 33, 35, 36, 38, 41, 45, 46, 51, 55, 56, 58, 60, 63, 65, 68, 71, 72, 73, 74, 78), anxiety (Table 1, nos: 1, 4, 5, 9, 15, 16, 19, 24, 31, 32, 33, 35, 36, 38, 46, 51, 55, 56, 58, 63, 68, 78), emotional/psychological distress (Table 1, nos: 3, 12, 15, 17, 20, 22, 26, 27, 28, 30, 43, 50, 64, 69, 70, 74, 76, perceived stress (Table 1, nos: 2, 31, 38, 45, 46, 52, 59, 67, 77), despair (Table 1, no. 69), negative mood/affect (Table 1, nos: 31, 32, 47, 53, 61, hostility (Table 1, no. 4), worry (Table 1, no. 75), cancer related intrusion (Table 1, no. 71) and loss of control (Table 1, no.: 46). Physical health states included: fatigue (Table 1, nos: 10, 12, 24, 25, 30, 56, 58, 63, 69), mortality (Table 1, no. 11), recurrence (Table 1, no. 11), physical health (Table 1, nos: 47, 64, 67), health related quality of life (Table 1, nos: 6, 18, 23, 28, 33, 39, 50, 52, 66), physical well-being (Table 1, no.: 54), somatic symptoms (Table 1, nos: 12, 38, 57), cancer related medical visits (Table 1, nos: 67, 70), functional status (Table 1, nos: 5, 42, 44), physical functioning (Table 1, Art.: 62), natural killer cell activity (Table 1, no. 44), pain (Table 1, nos: 4, 30, 60) and sleep (Table 1, no. 21).

### Definition and operationalization of potential moderators

The influence of cancer stage on the association between coping and health-related variables was examined by comparing a group of studies that included participants with breast cancer stage 0–II, here referred to as ‘early stage’, with a group of studies that included participants with breast cancer stage 0–IV, here referred to as ‘mixed stage’. The reason for not comparing stage 0–II with stage III–IV was that there were only two studies that exclusively included women at stage III or stage IV, while there were many studies that exclusively included participants at stage 0–II (Table 1). The influence of current cancer treatment on coping was assessed by comparing studies of women undergoing chemotherapy or radiation when coping was measured, with studies where the participants did not receive these kinds of treatments at that time. The influence of time since diagnosis was examined by comparing studies of samples of women diagnosed within the past six months at the time of measurement of coping with studies of women diagnosed earlier than the past six months. When examining the moderating effect of using situational or dispositional coping measures, we compared studies using a cancer-specific prompt with those using a dispositional prompt.

### Statistical methods

The meta-analysis was performed using Comprehensive Meta-Analysis software, Version 2.2.064. Bivariate correlation coefficients were used as measures of effect size. The correlations derived from the studies were Z-transformed before the meta-analysis by the formula [18]:
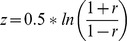



The results were then converted back to correlations for display. A random effects model was used to calculate effect sizes, since that model is generally more suitable than a fixed effect model when studies are gathered from the published literature and when the assumption is that true effect sizes differ between the studies [18]. Each study was weighted by the inverse of the within-study variance and the between-studies variance (Tau-Squared). If the p-value was ≤0.01, the correlations were regarded as significant. In the results, we interpreted effect sizes as small, medium, or large according to Cohen's convention for describing the strengths of correlations (small: *r* = 0.1, medium: *r* = 0.3, and large: *r* = 0.5) [19], [20]. Meta-analyses were performed for all possible combinations of coping and the health-related variables when there were at least two studies reporting such data. The results are presented in Tables 2–5. Publication bias was tested and corrected for using the trim-and-fill procedure described by Duval and Tweedie [21]. The corrected values are displayed in Tables 2–5. Heterogeneity between studies was assessed using the *Q* statistic. Potential interactions were tested if the correlation (effect size) was significant (p≤0.01), the *Q* statistic was significant at the level of 0.1 (p≤0.1) and there were at least two relevant studies at hand. Observed values related to heterogeneity are presented in Tables 2–5. The potential moderating influences of situational and measurement factors were assessed by testing interaction effect hypotheses. This was done by comparing effect sizes from the operationalized sub-groups, using the fixed effect model in which each study was weighted by the inverse of its variance. The fixed effect model was chosen since the subgroups in many cases included a very small number of studies, making the random effects model less suitable [18]. The chosen level of significance was here 0.01 (p≤0.01) and the observed interactions are displayed in Tables 2–4.

**Table 2:** Associations between coping and positive affect.

Coping scale	No. of studies	Total N	Mean *R*	Confidence interval	P	*R* adj. for publication bias (CI)	*Q* (p)	*I2*	Interactions
**Primary Control Coping**	17	3706	0.09	−0.04, 0.22	0.089		132.26 (<0.001)	87.903	
Direct Action	15	3323	0.15	0.18, 0.01	0.006		113.93 (<0.001)	87.712	TSD, CSC
Planning	4	437	−0.10	−0.37, 0.18	0.362		12.97 (0.005)	76.871	
Seeking Social Support	10	1466	−0.09	−0.26, 0.09	0.199		57.86 (<0.001)	84.446	
**Secondary Control Coping**	20	4238	0.28	0.15, 0.40	<0.001	0.26 (0.16, 0.36)	209.00 (<0.001)	90.909	CT, TSD
Acceptance	6	1072	0.22	0.07, 0.37	<0.001		17.34 (<0.004)	71.156	DS
Positive Reappraisal	14	3239	0.27	0.11, 0.41	<0.001		134.24 (<0.001)	90.316	TSD
Fighting Spirit	5	1043	0.20	−0.08, 0.45	0.066	0.24 (0.05, 0.41)	44.72 (<0.001)	91.055	TSD
**Disengagement Coping**	24	4589	−0.22	−0.32, −0.13	<0.001	−0.21 (−0.28, −0.14)	138.49 (<0.001)	83.393	CT, DS
Alcohol/Drug Disengagement	5	569	−0.26	−0.72, −0.15	<0.001	−0.32 (−0.41, −0.23)	4.64 (0.326)	13.766	
Behavioral Disengagement	10	1430	−0.19	−0.38, 0.01	0.014		74.88 (<0.001)	87.980	
Escape/Avoidance/Distancing	16	3591	−0.07	−0.19, 0.06	0.184		111.05 (<0.001)	86.492	
Hopelessness	6	1060	−0.42	−0.50, −0.34	<0.001	−0.45 (−0.51, −0.39)	7.15 (0.210)	30.051	
Social Isolation	0								
**Miscellaneous**									
Rumination	8	1146	−0.16	−0.37, 0.08	0.088		63.70 (<0.001)	89.011	
Self-Controlling	3	361	−0.02	−0.72, 0.71	0.960		60.56 (<0.001)	96.698	
Self-Blame	4	409	−0.36	−0.63, −0.00	0.009	−0.28 (−0.51, −0.02)	18.88 (<0.001)	84.106	
Spirituality	14	3201	0.06	−0.09, 0.21	0.282	0.09 (−0.02, 0.21)	114.61 (<00.01)	88.657	
Venting	9	1030	−0.07	−0.27, 0.14	0.399		48.00 (0.0)	83.334	

*Note*: CT  =  Current Treatment; CSC  =  Cancer Specific Coping; DS  =  Disease Stage (Cancer stage); TSD  =  Time Since Diagnosis.

**Table 3:** Associations between coping and negative affect.

Coping scale	No. of studies	Total N	Mean *R*	Confidence interval	P	*R* adj. for publication bias (CI)	*Q* (p)	*I2*	Interactions
**Primary Control Coping**	37	5549	−0.00	−0.07, 0.07	0.936	−0.06 (−0.11, −0.01)	115.64 (<0.001)	68.868	
Direct Action	31	4961	−0.02	−0.09, 0.04	0.315	−0.05 (−0.10, −0.00)	72.02 (<0.001)	58.343	
Planning	12	1176	0.10	−0.03, 0.22	0.045		28.04 (0.003)	60.763	
Seeking Social Support	23	2739	0.08	−0.04, 0.20	0.094		119.64 (<0.001)	81.612	
**Secondary Control Coping**	37	6122	−0.20	−0.27, −0.14	<0.001		133.62 (<0.001)	73.057	CT, TSD
Acceptance	15	1889	−0.24	−0.31, −0.16	<0.001	−0.21 (−0.26, −0.15)	19.27 (0.155)	27.352	
Positive Reappraisal	21	3776	−0.16	−0.25, −0.06	<0.001		86.03 (<0.001)	76.753	DS
Fighting Spirit	12	2520	−0.18	−0.29, −0.06	<0.001	−0.13 (−0.22, −0.03)	51.39 (<0.001)	78.595	TSD
**Disengagement Coping**	46	7109	0.24	0.17, 0.32	<0.001	0.17 (0.10, 0.23)	249.44 (<0.001)	81.96	CT, CSC
Alcohol/Drug Disengagement	11	1080	0.18	0.08, 0.27	<0.001		13.65 (0.190)	26.723	
Behavioral Disengagement	17	2660	0.17	0.04, 0.29	0.001		89.80 (<0.001)	82.183	CT, TSD
Escape/Avoidance/Distancing	35	5489	0.16	0.08, 0.23	<0.001	0.14 (0.08, 0.19)	136.26 (<0.001)	75.047	CT, CSC
Hopelessness	13	2557	0.41	0.22, 0.57	<0.001	0.33 (0.18, 0.46)	181.21 (<0.001)	93.378	TSD, DS, CSC
Social Isolation	1	80							
**Miscellaneous**									
Rumination	16	2869	0.39	0.25, 0.51	<0.001	0.32 (0.21, 0.43)	144.70 (<0.001)	89.634	
Self-Controlling	5	358	0.22	0.09, 0.35	<0.001	0.27 (0.18, 0.35)	2.12 (0.715)	0.000	
Self-Blame	9	650	0.38	0.24, 0.50	<0.001	0,32 (0.21, 0.43)	16.98 (0.030)	52.877	
Spirituality	18	3355	0.01	−0.07, 0.09	0.740	−0.03 (−0.09, 0.03)	38.34 (0.002)	55.666	
Venting	14	1437	0.19	0.03, 0.35	0.003		70.94 (<0.001)	81.675	TSD

*Note*: CT  =  Current Treatment; CSC  =  Cancer Specific Coping; DS  =  Disease Stage (Cancer stage); TSD  =  Time Since Diagnosis.

**Table 4:** Associations between coping and physical health.

Coping scale	No. of studies	Total N	Mean *R*	Confidence interval	P	*R* adj. for publication bias (CI)	*Q* (p)	*I2*	Interactions
**Primary Control Coping**	21	3971	−0.00	−0.11, 0.10	0.930	0.06 (−0.03, 0,14)	110.57 (<0.001)	81.913	
Direct Action	17	3354	0.03	−0.06, 0.12	0.475	0.09 (0.00, 0.18)	86.09 (<0.001)	81.415	
Planning	4	266	0.07	−0.26, 0.38	0.604		11.84 (0.008)	74.667	
Seeking Social Support	13	1821	−0.04	−0.16, 0.08	0.338	−0.11 (−0.20, −0.02)	39.74 (<0.001)	69.806	
**Secondary Control Coping**	15	2918	0.14	−0.07, 0.28	0.014		102.77 (<0.001)	86.377	CT
Acceptance	6	404	0.13	−0.18, 0.42	0.278		27.95 (<0.001)	82.111	
Positive Reappraisal	12	2395	0.18	−0.01, 0.36	0.017	0.20 (0.06, 0.34)	109.52 (<0.001)	89.956	
Fighting Spirit	2	465	0.04	−0.14, 0.22	0.567		1.755 (0.185)	43.011	
**Disengagement Coping**	26	4702	−0.12	−0.23, −0.02	0.002	-	170.83 (<0.001)	85.365	
Alcohol/Drug Disengagement	6	478	−0.05	−0.26, 0.17	0.562	−0.09 (−0.24, 0.07)	15.90 (0.007)	68.550	
Behavioral Disengagement	8	1098	−0.12	−0.32, 0.09	0.142		45.77 (<0.001)	84.707	
Escape/Avoidance/Distancing	18	3608	−0.06	−0.15, 0.03	0.078	−0.10, (−0.16, −0.03)	57.70 (<0.001)	70.538	
Hopelessness	7	1156	−0.22	−0.42, −0.00	0.009		45.60 (<0.001)	86.843	
Social Isolation	0								
**Miscellaneous**									
Rumination	4	703	0.05	−0.20, 0.30	0.618		17.43 (0.001)	82.793	
Self-Controlling	3	289	−0.12	−0.52, 0.33	0.512		15.18 (0.001)	86.821	
Self-Blame	4	237	−0.02	−0.48, 0.45	0.935		24.80 (<0.001)	87.903	
Spirituality	12	2555	0.03	−0.13, 0.20	0.613	0.08 (−0.06, 0.21)	93.00 (<0.001)	88.172	
Venting	7	518	−0.02	−0.24, 0.21	0.859	−0.11 (−0.28, 0.07)	23.52 (0.001)	74.496	

*Note*: CT =  Current treatment.

**Table 5:** Coping as a predictor of positive/negative affect and physical health in prospective studies.

	No. of studies	Total N	Mean *R*	Confidence interval	P	*R* adj. for publication bias (CI)	*Q* (p)	*I2*	Interactions
**Primary Control Coping**									
Outcome:									
Positive Affect	5	723	0.13	0.03, 0.22	0.001	0.14 (0.08, 0.21)	1.94 (0.747)	0.000	
Negative Affect	5	686	0.03	−0.10, 0.16	0.524	−0.04 (−0.15, 0.06)	5.35 (0.253)	25.240	
Physical Health	2	203	−0.02	−0.12, 0.16	0.796		<0.01 (0.967)	0.000	
**Secondary Control Coping**									
Outcome:									
Positive Affect	6	1412	0.17	0.08, 0.28	<0.001	0.22 (0.14, 0.31)	8.79 (0.118)	43.105	
Negative Affect	3	535	−0.19	−0.38, 0.01	0.014		4.10 (0.129)	51.198	
Physical Health	4	929	−0.06	−0.38, 0.27	0.641	0.01 (−0.20, 0.21)	26.15 (0.001)	85.128	
**Disengagement Coping**									
Outcome:									
Positive Affect	8	1593	−0.19	−0.37, −0.04	0.002	−0.23 (−0.36, −0.08)	46.37 (<0.001)	84.904	DS
Negative Affect	6	725	0.24	0.04, 0.43	<0.002	0.20 (0.04, 0.35)	16.7 (0.005)	70.056	
Physical Health	5	1045	−0.14	−0.29, 0.03	0.032		9.85 (0.043)	59.388	

*Note*: DS  =  Disease Stage.

## Results

### Study sample characteristics

A total of 78 studies and 11 948 participants were included in the final analyses. 62 studies only presented cross-sectional correlations, while 16 reported on correlations between baseline assessments of coping styles and follow-up measures of psychological or physical states. Ten studies included both cross-sectional and prospective data. The studies and participant characteristics are presented in Table 1. Sample sizes ranged from 32 to 1083. The mean age of study participants was 53 years, and study means ranged from 43 to 62 years. Only 26 studies reported on mean time since cancer diagnosis, with an average of 1.8 years. However, 30 studies reported results from women recruited within 6 months of diagnosis, 22 reported results from women recruited more than 6 months after diagnosis, and 25 reported a mix of patients recruited at various times since diagnosis. The majority of studies included women with no previous cancer diagnosis, but two reported on results from participants with recurrent disease. Nine studies reported on a mix of first and recurrent diagnoses, while 21 did not report details of previous cancer experience. The scales used to measure coping are presented in Table 1. 32 studies used some version of the COPE scale, ten used Ways of Coping (WOC), ten used a version of the Mental Adjustment to Cancer (MAC) scale, while the remaining studies used other scales. 51 studies specified coping with cancer in their measures, 12 asked about other problems or general use of coping strategies, and 9 did not specify a specific stressor. 40 studies reported on measures of positive affect, 56 on measures of negative affect, and 33 on measures of physical health outcomes.

### Lower-order coping categories and psychological and physical states

Results of the meta-analyses of associations between coping and positive affect are presented in Table 2. Direct Action, Acceptance and Positive Reappraisal were associated with higher levels of positive affect, with Positive Reappraisal showing the strongest correlation. Alcohol/Drug Disengagement, Hopelessness, and Self-Blame were associated with lower positive affect. The majority of the effects were small to medium, but the effect for Hopelessness tended towards large [19]. Possible publication bias was apparent for Alcohol/Drug Disengagement, Hopelessness and Self-Blame, but the associations remained significant after trim-and-fill adjustment. Publication bias was also tested for coping strategies with non-significant associations with positive affect. After trim-and-fill adjustment, the association between Fighting Spirit and higher positive affect became significant, showing a small to medium effect. Spirituality also showed signs of publication bias, but the association was still non-significant after trim-and-fill adjustment. The results of the meta-analyses of the associations between coping and negative affect are presented in Table 3. Acceptance, Positive Reappraisal, and Fighting Spirit were associated with lower levels of negative affect, with Acceptance showing the strongest correlation. Alcohol/Drug Disengagement, Behavioral Disengagement, Escape/Avoidance/Distancing, Hopelessness, Rumination, Self-Controlling, Self-Blame, and Venting were associated with higher negative affect. Most of the effects were small to medium. Tests for publication bias showed possible bias for Acceptance, Fighting Spirit, Escape/Avoidance/Distancing, Hopelessness, Rumination, Self-Blame, and Self-Controlling. After trim-and-fill adjustment, the associations remained significant. Tests for publication bias were also made for Direct Action and Spirituality, which both showed non-significant associations with negative affect. Indications of publication bias were shown for both, and after trim-and-fill adjustment, the association between Direct Action and negative affect became significant with a weak association with lower levels of negative affect; but the association between Spirituality and negative affect remained non-significant. Results of the meta-analyses of the associations between coping and aspects of physical health are presented in Table 4. Hopelessness was associated with lower levels of physical health, with a small to medium effect size. Indications of publication bias were found for Direct Action, Seeking Social Support, Positive Reappraisal, Alcohol/Drug Disengagement, Escape/Avoidance/Distancing, Spirituality, and Venting. After trim-and-fill adjustment, Direct Action became significantly, albeit weakly, associated with higher levels of physical health and Positive Reappraisal showed a significant association to higher level of physical health with a small to medium effect size. Seeking Social Support and Escape/Avoidance/Distancing became significant, in that they were weakly associated with lower levels of physical health after trim-and-fill adjustment. The other associations remained non-significant.

### Higher-order coping categories and psychological and physical states

Using the higher-order coping categories, meta-analyses were performed on the three health-related variables. Five of the lower-order coping strategies (Rumination, Self-Controlling, Self-Blame, Spirituality, and Venting) did not clearly fall into any of the higher-order categories and were therefore excluded. The results are presented in Tables 2−4. Primary Control Coping (PCC) was unrelated to both positive and negative affect, and to physical health. Possible publication bias was shown for PCC and negative affect, and also for PCC and physical health. After trim-and-fill adjustment, the association between coping and negative affect became significant, with a small effect size, while the association between PCC and physical health remained non-significant. Secondary Control Coping (SCC) was significantly related to higher positive affect and lower negative affect but unrelated to physical health. Tests for publication bias indicated possible bias for SCC and positive affect. After trim-and-fill adjustment, the association remained significant with an effect tending towards medium. Disengagement Coping (DC) was significantly related to lower positive affect, higher negative affect, and lower physical health. The effect sizes here were small to medium. Tests for publication bias indicated possible bias for positive and negative affect. After trim-and-fill adjustment, the associations remained significant.

### Coping as a predictor in prospective studies

Due to the small number of studies reporting correlations between baseline assessment of coping and follow-up measures of health-related variables, we were only able to perform prospective meta-analyses on the basis of the higher-order coping strategies. 14 studies and 2,073 participants were included in these analyses. The mean follow-up time after baseline was 1.5 years. The results are presented in Table 5. PCC was, by contrast with the results of the cross-sectional analysis, found to be positively related to positive affect. But, in accordance with the results of the cross-sectional studies, it was found to be unrelated to negative affect and to physical health. Tests for publication bias indicated possible bias for PCC and positive and negative affect. After trim-and-fill adjustment, the association between PCC and positive affect was still significant, and the association between PCC and negative affect still non-significant. SCC was, in line with the cross-sectional studies, found to be positively related to positive affect, but unrelated to negative affect and physical health, although the p-value for negative affect was quite low (P = 0.014). The association between SCC and positive affect was weaker in the prospective analysis, as compared with the cross-sectional analysis. A test for publication bias indicated possible bias for SCC and positive affect and physical health. After trim-and-fill adjustment, the association remained significant for positive affect and non-significant for physical health. Like in the cross-sectional studies, DC was negatively related to positive affect and positively related to negative affect, but unrelated to physical health. The effect sizes were similar to those in the cross-sectional analyses. Tests for publication bias showed possible bias for DC and positive and negative affect. After trim-and-fill adjustment, the associations remained significant.

### Test of moderation

The Q statistics and the results of the testing of interactions are presented in Tables 2−4.

### Cancer stage

Two of the SCC strategies showed interactions with cancer stage. The positive association between Acceptance and positive affect was stronger among women with mixed-stage breast cancer, as compared with women with early-stage breast cancer (interaction p = 0.001; Stage 0−II r = 0.12, p = 0.003; Stage 0−IV r = 0.34, p<0.001). To the contrary, the negative association between Positive Reappraisal and negative affect was slightly stronger among women with early-stage breast cancer, as compared with women with mixed-stage breast cancer (interaction p<0.001; Stage 0−II r = −0.23, p<0.001; Stage 0−IV r = −0.11, p<0.001). There was no interaction regarding cancer stage in the higher-order category of SCC that could confirm or contradict these results. Some interactions were found among the DC strategies. Hopelessness was more strongly related to higher levels of negative affect in the mixed-stage group (r = 0.44, p<0.001), as compared with in the early-stage group (r = 0.26, p<0.001; interaction p<0.001). The result was highlighted by an interaction between the higher-order DC category and positive affect, showing that DC was more strongly related to lower positive affect in the mixed-stage group (r = −0.29, p<0.001) than in the early-stage group (r = −0.13, p<0.001; interaction p<0.001). The prospective analysis further supported this pattern, since there was a negative association between Disengagement Coping and positive affect only in the mixed-stage breast cancer group (r = −0.19, p<0.001; interaction p = 0.003).

### Current treatment

No interactions regarding current treatment were found among lower-order coping strategies in the PCC and SCC categories. However, the higher-order SCC was more strongly related to higher positive affect among women undergoing treatment (r = 0.47, p<0.001) than among women not undergoing treatment (r = 0.10, p = 0.008; interaction p<0.01), and more strongly related to lower negative affect among the treated (r = −0.27, p<0.001) than among the non-treated (r = −0.13, p<0.001; interaction p = 0.002). Further, SCC was only related to higher physical health among women undergoing treatment (r = 0.42, p<0.001; interaction p<0.001). The test of current treatment as a potential moderator regarding more disengagement forms of coping showed that Behavioral Disengagement was significantly associated with higher negative affect only in the group of women under current treatment (r = 0.35, p<0.001; interaction p<0.001). In line with this, Escape/Avoidance/Distancing was only associated with higher negative affect among the treated (r = 0.24, p<0.001) than the non-treated (r = 0.08, p<0.006; interaction p = 0.001). These results are in line with analyses of the interactions at the higher level of DC, which show a stronger association with higher negative affect among treated women (r =  0.41, p<0.001) than among those not treated (r = 0.14, p<0.001; interaction p<0.001), and a significant association with lower positive affect only among treated women (r = −0.33, p<0.001; interaction p<0.001).

### Time since diagnosis

The association between the Direct Action PCC strategy and higher positive affect was significant only among newly diagnosed women (r = 0.21, p<0.001; interaction p<0.001). Further, the association between the Positive Reappraisal SCC strategy and higher positive affect was stronger among newly diagnosed women (r = 0.24, p<0.001) than among women diagnosed earlier than during the past six months (r = 0.11, p<0.001; interaction p = 0.003). The SCC strategy Fighting Spirit showed an association with lower negative affect only among newly diagnosed women (r = −0.22, p<0.001; interaction p<0.001) and an association with higher positive affect only among newly diagnosed women (p = 0.31; interaction p<0.001). In line with this, the higher order SCC was more strongly related to higher positive affect among newly diagnosed women (r = 0.36, p<0.001) as compared to those diagnosed at an earlier time-point (r = 0.12, p<0.001; interaction p<0.001) and also related to less negative affect among newly diagnosed women (r = −0.24, p<0.001), as compared with those diagnosed at an earlier time-point (r = −0.12, p<0.001; interaction p<0.001). The DC Hopelessness category showed a slightly weaker association with higher negative affect in the group of newly diagnosed women (r =  0.23, p<0.001) as compared with women diagnosed earlier (r = 0.46, p<0.001; interaction p<0.001). To the contrary, the association between the Behavioral Disengagement DC strategy and higher negative affect was stronger among the newly diagnosed women (r = 0.23, p<0.01) than among the women diagnosed earlier (r = 0.09, p<0.01; interaction p = 0.004). The association between Venting and higher negative affect was slightly stronger among the newly diagnosed (interaction p = 0.007; ≤6 months r = 0.41, p<0.001;>6 months r = 0.21, p<0.001).

### Situational or dispositional assessment

The association between the Direct Action PCC strategy and higher positive affect was present only in the group of studies using a dispositional prompt for the measuring of coping (r = 0.31, p<0.001; interaction p<0.001). No other interactions were observed among either PCC strategies or SCC strategies. Among DC strategies we found two interactions at the lower level. The association between Escape/Avoidance/Distancing and higher negative affect was significant only in the group of studies using a cancer-specific prompt (r = 0.21, p<0.001; interaction p<0.001), and Hopelessness showed a correlation with higher negative affect only when using a cancer-specific prompt (r = 0.39, p<0.001; interaction p = 0.002). The analyses at the higher level were partially in line with these results. The association between DC and higher negative affect was significant only when using a cancer-specific prompt (r = 0.28, p<0.001; interaction p<0.001).

## Discussion

In the current study, we have analyzed coping strategies using a structure with lower-order and higher-order categories. The use of higher-order coping categories was a way of facilitating the interpretation of findings, but lower-order coping categories can give more valuable information relevant to the development of interventions to promote effective coping. Overall, and similar to earlier meta-analytic findings, more engagement forms of coping, aiming to eliminate, reduce, or manage stressors or their emotional consequences, were found to be related to better psychological and physical states than more disengagement forms of coping, aiming to avoid, ignore, or withdraw from stressors or their emotional consequences [14], [22], [23]. But, importantly, our findings gave stronger support for SCC strategies as compared with PCC strategies. By contrast with previous studies, indicating, for example, that the lower-order Direct Action PCC strategy is connected with better psychological and physical states [14], we found only a weak connection between Direct Action and positive affect, and no, or only very weak associations between Direct Action and negative affect and physical health respectively. That said, the results of the interaction analyses suggest that Direct Action is more effective among newly diagnosed women, as compared with women with a longer history of disease; and having a general disposition towards Direct Action seems to be more beneficial than adopting Direct Action as a cancer-specific coping strategy. Planning, which is another lower-order PCC strategy, involves thinking about how to confront a stressor, and planning one's active coping efforts to counter stressful experiences. This strategy was found to be unrelated to both positive and negative affect and physical health. Despite the generally weak effects of PCC strategies, our study did give some support for a beneficial effect of PCC in handling stressful situations. We found a weak but significant association between PCC and positive affect over time in the prospective studies. However, strategies based on Secondary Control Coping (SCC) were found to be more strongly related to positive psychological states in both the cross-sectional and prospective studies. Further, Acceptance appeared to be more beneficial in studies with mixed-stage breast cancer patients than in studies of women with early-stage breast cancer. Positive Reappraisal, on the other hand, appeared to be more beneficial for women with early-stage breast cancer. Fighting Spirit appeared to be more beneficial for newly diagnosed women. The analysis of higher-order SCC was also in line with these findings. Interaction analysis of treatment and higher-order SCC further suggested that women in treatment for cancer seem to obtain greater benefit from this type of strategies than those not treated. The beneficial effects of Secondary Control Coping shown in the cross-sectional studies are in line with the findings of the prospective studies, giving support for encouraging adaptation and adjustment to a situation or associated stressful emotions among women with breast cancer, rather than for putting effort into managing and directly controlling the stressors themselves. These findings are in line with results from several recent intervention studies promoting acceptance and non-reactivity among cancer patients [24]−[28]. The studies show promising results in relation to reducing psychological distress, and also to indicators of increased physical health. All of the Disengagement Coping (DC) strategies include different ways of distancing oneself from the stressor or related feelings, and thus the giving-up of efforts to control or adjust to a situation and associated emotions. All of the coping strategies analyzed and classified as DC were either related to lower positive affect and higher negative affect, or unrelated to the health-related variables. The analyses showed that DC seems to be more or less maladaptive depending on situation. For women treated for breast cancer, disengagement forms of coping seem to be more maladaptive than for women not undergoing treatment. Further, disengagement coping seems to be more maladaptive among women with mixed-stage breast cancer than among those with early-stage breast cancer. In addition to the coping strategies classified as DC, a few other types of coping appeared to be maladaptive in most of the analyses. Although few studies reported on Self-Blame, this strategy was among the types of coping most strongly related to lower positive affect and higher negative affect. Self-Controlling and Rumination were both related to higher negative affect. Self-Blame refers to efforts to place part of the causal responsibility for a situation or circumstance on oneself, while Self-Controlling refers to efforts to keep one's feelings to oneself. Rumination refers not only to the process of giving careful thought to something, but also to the tendency to go over things in one's mind repetitively and often negatively. Rumination has been associated with several negative outcomes, such as depressive symptoms, negative affect, poor problem-solving, and increased stress-related problems [29]. Our meta-analysis confirmed such deleterious effects of Rumination in dealing with stressors. Venting also relates to coping through an increased awareness of one's emotional distress, and in particular to letting one's feelings out. Similar to Rumination, our meta-analyses showed Venting to be associated with higher negative affect. Such maladaptive effects of Rumination and Venting as ways of coping with stressful situations are important, given the emphasis placed on emotional processing and expression as elements in some of the psychosocial interventions offered to cancer patients [30], [31], and also in those theories of emotion that suggest that expression and exploring of stress-related emotions are beneficial. Although we could not examine the predictive value of these coping strategies in relation to future psychological functioning in prospective studies, the cross-sectional studies indicate associations between them and maladaptive outcomes. Spirituality was found to be unrelated to positive and negative affect as well as to physical health. A great number of studies have tried to show the beneficial effects of Spirituality as a way of coping with stressful situations and related emotions, but there has been little consistency in findings on the influence of different types of religious and spiritual coping on psychological well-being. Our findings are in line with the results of a systematic review of the influence of religion and spirituality on psychological well-being [32]. The review concludes that there is no support for a positive effect of religious or spiritual coping on psychological well-being; to the contrary, it seems to have a detrimental effect on well-being over time in some circumstances.

### Limitations

While this study broadens previous knowledge in the coping field by summarizing the rather wide and heterogeneous nature of the research, there are several limitations to our analysis and to interpretations of the importance of results. First, searching in only two databases may have resulted in the exclusion of some relevant articles, even though we found a fairly large number of studies. Second, the large majority of studies were cross-sectional, thereby making it impossible to draw conclusions regarding the directions of associations. It is likely that psychological and physical functioning are outcomes that influence type of coping as well as being outcomes that are influenced by type of coping. A longitudinal study from 2009 supports the notion of reciprocal relationships between coping strategies and psychological functioning, in that it shows that measures of quality of life can predict future coping strategies [33]. We were not able to assess such more complex relations between coping and outcomes. Third, a fairly large number of different scales and measures of coping were used in the studies included, and they needed to be placed into more manageable groups. In the process, a small number of coping strategies were left out, and some others may have been misclassified. In the articles, the scales were not always described in detail, and the classifications were sometimes made using a few sample items, on some occasions using only the name of the coping sub-scale. Fourth, our calculation of mean scores for different health-related variables might have lowered precision, and led to the mixing of some quite different measures into one. However, given the number of studies available and the efforts made to bring greater clarity to our findings, we judge our summations of estimated values to be necessary and reasonable. Fifth, only studies from Western countries were assessed in the analysis. This may have led to biased results with regard to ethnic factors and thus limiting the generalizability of the findings. Future research could help clarify if associations between coping and health in non-Western populations are consistent with, or differ from those among non-Western populations. Lastly, we were unable clearly to examine differences in coping among patients with early- and later-stage disease. However, we were able to distinguish some differences in coping between women with early-stage disease and those used in samples of mixed-stage cancer patients.

Although the research examining what works in coping with cancer is extensive, with a large number of publications reporting on different aspects of coping and cancer, there is a need for more studies to extend our understanding, and to develop a more clearly defined theoretical framework. The theoretically based higher-order categorization of coping strategies into primary control engagement coping, secondary control engagement coping, and disengagement coping was supported in this study, and should be considered in future studies. To facilitate comparisons of results in future studies, standardized and well-used measurement scales for coping, such as the Ways of Coping and the COPE, should be employed. If necessary, additional items might be added to expand the breadth of coping strategies measured on these scales. The quality of future studies would also benefit from using longitudinal designs, and from the more uniform reporting of results. In particular, univariate statistics on associations between coping measures and health-related variables should be reported, as too should statistics controlling for relevant covariates.

## Conclusions

This meta-analysis indicates that efforts to facilitate adaptation to stress, such as Acceptance and Positive Reappraisal, are particularly beneficial in coping with stressors related to breast cancer. Disengagement and avoidance types of coping seem to be consistently maladaptive in dealing with breast-cancer-related stressors, and are associated with lower psychological functioning and physical health. The study further indicates that, in several circumstances, coping effectiveness is dependent on situational and measurement factors. Treatment appears to strengthen the associations with both engagement and disengagement forms of coping, which suggests that women undergoing treatment for their cancer are particularly likely to benefit from replacing disengagement forms of coping with more adaptive coping strategies.

## Supporting Information

Checklist S1 **PRISMA 2009 Checklist.**
(DOC)

Data S1 **Data underlying the meta-analysis.**
(XLSX)

References S1 **References included in the meta-analysis.**
(DOC)
