# Supplementary material for: Coping with Breast Cancer: A Meta-Analysis
Source: PLoS One. 2014 Nov 25;9(11):e112733. doi: 10.1371/journal.pone.0112733 (PMC4244095; doi:10.1371/journal.pone.0112733)
Supplement: References S1 — References included in the meta-analysis. (DOC) [file pone.0112733.s003.doc]

**References included in the meta-analysis**
